# Decrypting the Potential of Nanotechnology-Based Approaches as Cutting-Edge for Management of Hyperpigmentation Disorder

**DOI:** 10.3390/molecules28010220

**Published:** 2022-12-26

**Authors:** Sukhbir Singh, Neelam Sharma, Ishrat Zahoor, Tapan Behl, Anita Antil, Sumeet Gupta, Md Khalid Anwer, Syam Mohan, Simona Gabriela Bungau

**Affiliations:** 1 Department of Pharmaceutics, MM College of Pharmacy, Maharishi Markandeshwar (Deemed to Be University), Mullana, Ambala 133207, Haryana, India; 2 Chitkara College of Pharmacy, Chitkara University, Rajpura 140401, Punjab, India; 3 School of Health Sciences &Technology, University of Petroleum and Energy Studies, Bidholi, Dehradun 248007, Uttarakhand, India; 4 Janta College of Pharmacy, Butana, Sonipat 131302, Haryana, India; 5 Department of Pharmacology, MM College of Pharmacy, Maharishi Markandeshwar (Deemed to Be University), Mullana, Ambala 133207, Haryana, India; 6 Department of Pharmaceutics, College of Pharmacy, Prince Sattam Bin Abdulaziz University, Alkharj 16278, Saudi Arabia; 7 Substance Abuse and Toxicology Research Centre, Jazan University, Jazan 45142, Saudi Arabia; 8 Center for Transdisciplinary Research, Department of Pharmacology, Saveetha Dental College, Saveetha Institute of Medical and Technical Science, Saveetha University, Chennai 602117, Tamil Nadu, India; 9 Department of Pharmacy, Faculty of Medicine and Pharmacy, University of Oradea, 410028 Oradea, Romania; 10 Doctoral School of Biomedical Sciences, University of Oradea, 410087 Oradea, Romania

**Keywords:** Fitzpatrick skin phototype, melasma, melanin, nanocarriers, hyperpigmentation, melanocytes

## Abstract

The abundant synthesis and accretion of melanin inside skin can be caused by activation of melanogenic enzymes or increase in number of melanocytes. Melasma is defined as hyperpigmented bright or dark brown spots which are symmetrically distributed and have serrated and irregular borders. The three general categories of pigmentation pattern include centro facial pattern, malar pattern, and mandibular pattern. Exposure to UV rays, heat, use of cosmetics and photosensitizing drugs, female sex hormonal therapies, aberrant production of melanocyte stimulating hormone, and increasing aesthetic demands are factors which cause the development of melasma disease. This review gives a brief overview regarding the Fitzpatrick skin phototype classification system, life cycle of melanin, mechanism of action of anti-hyperpigmenting drugs, and existing pharmacotherapy strategies for the treatment of melasma. The objectives of this review are focused on role of cutting-edge nanotechnology-based strategies, such as lipid-based nanocarriers, i.e., lipid nanoparticles, microemulsions, nanoemulsions, liposomes, ethosomes, niosomes, transfersomes, aspasomes, invasomes penetration-enhancing vesicles; inorganic nanocarriers, i.e., gold nanoparticles and fullerenes; and polymer-based nanocarriers i.e., polymeric nanoparticles, polymerosomes, and polymeric micelles for the management of hyperpigmentation.

## 1. Introduction

Hyperpigmentation is attributed to melanocytes which generates excess amounts of melanin, which is subsequently captivated through keratinocytes and accumulated within dermis [1,2]. Melasma is a highly prevalent and globally recurrent disorder of hyperpigmentation. Melasma is derived from the Greek word “melas”, which means “black”, and refers to the clinical appearance of dark brownish spots. This disorder is also referred to as “chloasma”, which means “green in hue” [3]. Melasma involves symmetrically distributed pigmentation with sharply bordered light brown to bluish-grey patches and freckle like spot with uneven and spiky borders which develops gradually over weeks or years [4]. In wintertime, hypepigmentation patches gets often fade but appears worse in summer. Pigmentation might be in the form of spots, confetti, linear, or continuous lines. The three major patterns of pigmentation distribution include: (i) centro facial pattern, which has been observed in 65% of cases and affects cheeks, forehead, upper lip, and nose; (ii) malar pattern, which specifically affects the cheeks as well as nose and has been identified in 20% of cases; and (iii) mandibular pattern, which has been recognized in 15% of the cases and distinctively affects mandibular ramus [5,6]. Furthermore, melasma is most typically described in females in their twenties and thirties during their reproductive years. Melasma is uncommon in men, who account for less than 10% of all instances. It is rarely recorded before the age of adolescence. Melasma over extra-facial region is frequently observed in postmenopausal women [7]. The melasma is a photoageing condition in genetically susceptible people and has a detrimental influence on the patient’s quality of life [8]. The Fitzpatrick skin phototype classification system, life cycle of melanin, mechanism of action of anti-hyperpigmenting drugs, and pharmacotherapy strategies for the treatment of melasma are described in this review. The objectives of this review are focused on an overview of the role of lipid-based, inorganic, and polymer-based nanocarriers for the management of melasma. For this purpose, an extensive search of the literature was conducted using the Google Scholar, PubMed, and ScienceDirect databases from year 2000 to 2022.

## 2. Fitzpatrick Skin Phototype (FSPT) Classification System

With the help of light induced fluorescence, Wood’s light may be used to assess the depth of melanin. Based on the Wood’s light examination, melasma is divided into four clinical types, i.e., epidermal, dermal, dark-brown, and indeterminate or inapparent melasma. Epidermal melasma appears as light brown, and under Wood’s light, strong pigmentation is observed when melanin is spread across epidermal layers. The dermal melasma gets manifested as brown or blue grey colored patches under visible light along with a lot of pigmented cutaneous melanophages. In dark-brown melasma, Wood’s lamp light might promote pigmentation enhancement in certain regions while leaving others unchanged. Indeterminate or unapparent melasma mainly affects dark-brown skinned people [7,8,9]. The FSPT system was based upon the measurement of sun sensitivity and is specifically used to evaluate sunburn and suntan risk of patients. According to FSPT, individuals and races differ in their vulnerability to sunlight, as shown in Table 1 [8,10,11,12].

## 3. Life Cycle of Melanin

**Figure 1:**
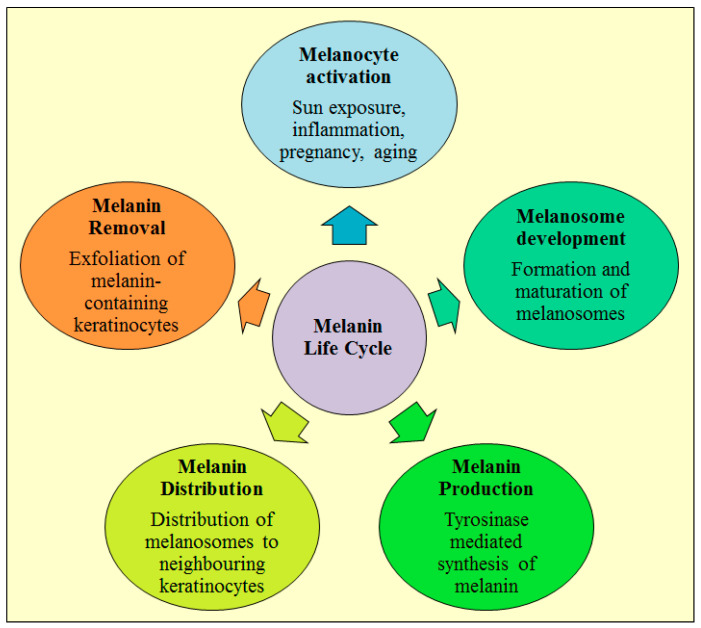
Various stages of melanin life cycle (i) melanocyte activation, (ii) melanosome development, (iii) melanin production, (iv) melanin distribution and (v) melanin removal.

Hemoglobin, melanin, and carotene are the pigments which determine the skin color. The epidermal elements are involved in melanogenesis process which involves the production of melanin in melanosomes by melanocytes and gets deposited throughout epidermis [9,13,14]. Melanogenesis is induced through paracrine interaction between keratinocytes and melanocytes which expedite the release of melanogenic factors like pro-opiomelanocortin-derived peptides. The melanocyte present in epidermis stratum basale generates melanin and delivers to keratinocytes which subsequently combine, aggregates, and degrades resulting in change of skin color [15,16,17]. Melanocytes are skin pigmentation cells that contribute to melanin and have five stages during its lifecycle (Figure 1). Various compounds found in many skin care products have the potential to inhibit some of steps of melanin life cycle. Melanocytes generate melanin and subsequently transport melanin to the keratinocytes in epidermis. Melanin is produced when some proteins have been relocated and sorted to melanosomes [18]. The melanin generation is mediated through tyrosinase enzyme and pigmentation problems manifest in either of two ways, i.e., hyperpigmentation (rise in skin pigmentation) or hypopigmentation (decrease in skin pigmentation). Melasma (chloasma) and ephelides (freckles) are pigmentation disorders which are linked to skin pigmentation disturbances. Melasma can be caused by epidermal melanocyte hyperactivity which can lead to an increase in pigmentation [19,20,21,22,23].

## 4. Pharmacotherapy Approaches for Management of Hyperpigmentation

**Figure 2:**
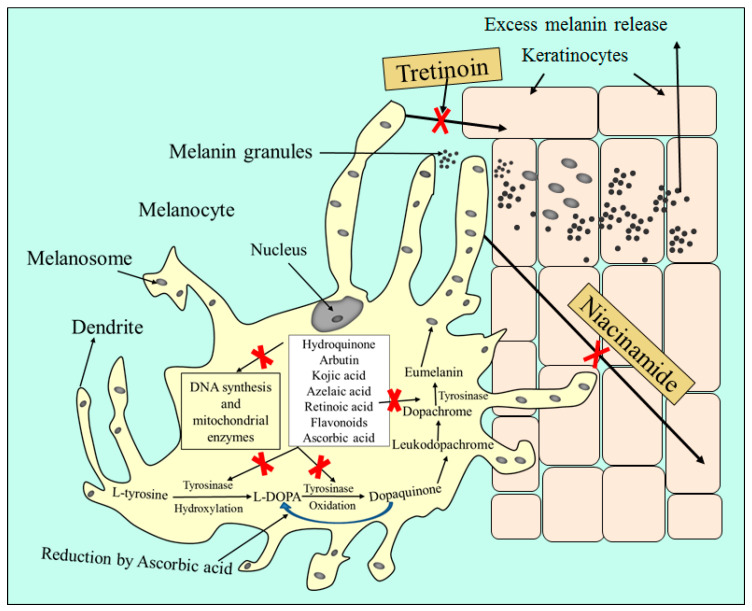
The mechanism of action of anti-hyperpigmentation drugs via topical application.

The primary crucial step in determining efficient and appropriate treatment for melasma patient is to determine the severity of melasma. Physical therapy with intense pulse light sources or lasers, chemical peels, dermabrasion, and microneedle technology are a few treatment strategies for melasma. Melasma can be treated with variety of topical and systemic drug administration. Oral therapies for the management of melasma include cyklokapron (tranexamic acid), glutathione, and polypodium aureum extract obtained from tropical fern leaves. Drugs, such as hydroquinone, kojic acid, azelaic acid, retinoic acid, flavonoids, arbutin, and ascorbic acid, are some examples which have potential activity for the topical therapy of hyperpigmentation [4]. Various photoprotective precautions include avoiding direct sun exposure and applying sunscreen on a regular basis should always be encouraged [9]. The mechanisms of action of drugs for management of hyperpigmentation via topical application are depicted in Figure 2.

## 5. Role of Lipid-Based Nanocarriers in Hyperpigmentation

**Figure 3:**
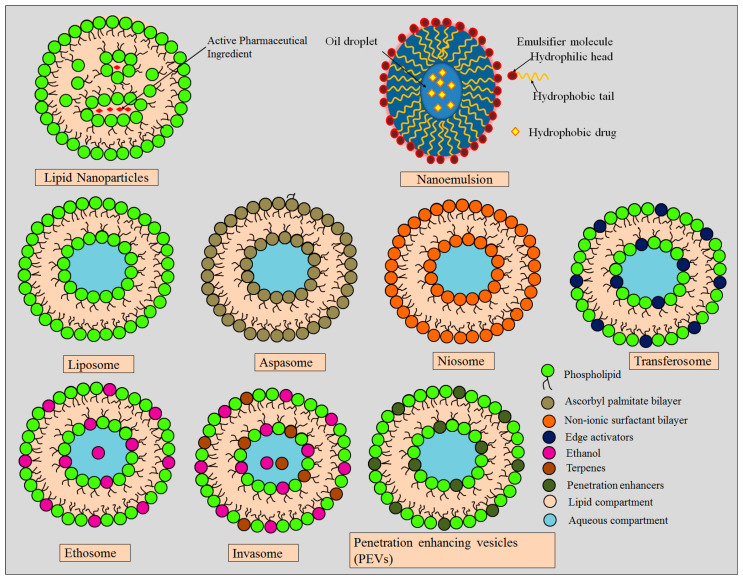
Structural representation of various lipid based nanocarriers.

Nanotechnology based treatment strategies have immense potential to improve therapeutic potential of anti-hyperpigmenting drugs for exclusive therapy of hyperpigmentation. The encapsulation of topical hypopigmenting drugs within nanocarriers-based delivery systems has been frequently explored recently for the effective management of melasma. These nanocarrier strategies have numerous advantages which include enhanced drug permeation, drug targeting, improved therapeutic potential, stability against degradation, as well as rapid and prolonged action [24,25]. A diagrammatic representation of various lipid based nanocarriers is depicted in Figure 3.

### 5.1. Lipid Nanoparticles

Lipid nanoparticles (LNPs) are novel pharmaceutical delivery systems which include nanostructured lipid carriers (NLCs) and solid lipid nanoparticles (SLNs) [26]. SLNs are spherical particles comprised of solid lipid having average diameter of 10–1000 nm and exists in solid state at ambient temperature [27]. NLCs are comprised of physiological and biocompatible solid lipid and liquid lipid, surfactants, and co-surfactants. NLCs have increased storage stability and loading capacity along with a reduced probability of drug leakage [28]. LNPs have potential applications in cosmetic and dermatological formulations, e.g., supporting improvements in skin elasticity, hydration, permeation, and drug targeting [29].

### 5.2. Microemulsion and Nanoemulsion

Microemulsions are homogenous, transparent, thermodynamic, and stable dispersions having droplet diameter in the range of 10–100 nm in which the surfactant coating serves as barrier between oil and water phases [30,31,32]. ME has capability to permeate hydrophilic as well as lipophilic drug molecules as liquid membrane carriers [33]. Microemulsion can increase the rate at which moisturizing chemicals or substances are transferred into skin. Microemulsion has the potential to facilitate the permeation of a drug into deeper skin layers by interrupting the stratum corneum lipid’s organized structure which results in loss of skin’s barrier characteristics which facilitates drug transport [34].

Nanoemulsion has been defined as an ultrafine homogeneous transparent or translucent oil-in-water or water-in-oil emulsion with mean droplet diameter ranging from 20 to 200 nm which has been stabilized by surfactants [35,36,37,38]. It can be prepared under high mechanical extrusion process through mixing oil phase with aqueous phase [39,40,41]. Nanoemulsion has potential to solubilize poorly soluble drugs and enhance the drug penetration across skin layers via topical administration [42,43].

### 5.3. Liposomes

Liposomes are lipid bilayer vesicles composed of phospholipids having 50–1000 nm diameter and serve as transport vehicles for physiologically active hydrophilic and lipophilic molecules [44,45]. These vesicular systems impart several advantages such as augmentation of drug’s solubility, bioavailability and stability. Furthermore, liposomes have great possibility for coupling with ligands to acquire active targeting to increase and maintain the therapeutic activity of drugs for prolonged period of time [45,46,47]. Liposomes are classified as small unilamellar vesicles (SUVs), large unilamellar vesicles (LUVs), and multilamellar vesicles (MLVs) with diameter of 100 nm, 200–800 nm, and 500–5000 nm, respectively [48]. Liposomes are good carriers for topical drug delivery because of their capability to prevent drug degradation and to encapsulate lipophilic as well as hydrophilic drugs. These also have strong affinity for keratin in skin’s horny layer and can penetrate deeper into skin. Liposomes tends to improve solubilization of poorly soluble drugs, act as permeation enhancer, have potential to produce local depot and tends to reduce adverse effects of drug [49,50]. Shigeta et al. found that the loading of linoleic acid into liposomes increased its solubility and hypopigmenting efficacy compared to non-liposomal preparations. Linoleic acid is adipose acids with concomitant inhibitory action on tyrosinase [51]. Another research study found that liposome production of anthocyanin (flavonoid with antioxidant and tyrosinase inhibitory properties) improved its photoprotective and antityrosinase activity in comparison to plain drug [52]. The encapsulations of *Aloe vera* extract (containing aloesin) into liposomes exhibited greater bioavailability and provided improved skin hypopigmenting activity as compared to pure extract [53]. Huh et al. described that the encapsulation of 4-n-butyl resorcinol into liposomes improved the stability and skin penetrability along with increase in tyrosinase inhibitory action to produce greater melanogenesis inhibitory effect [54]. *Asparagus racemosus* extract loaded liposomes suppressed the tyrosinase enzyme activity significantly in comparison to plain extract [55]. The liposomes production of phenylethyl resorcinol improved solubility, physical stability, and anti-tyrosinase action in comparison to plain drug [56]. *Artocarpus lakoocha* extract loaded liposomes exhibited improved skin penetration and skin whitening potential as compared to non-encapsulated extract [57]. In another investigation, it was found that the incorporation of arbutin into liposomes exhibited enhanced accumulation of arbutin in epidermis/dermis layers and improved the skin whitening potential [58].

### 5.4. Ethosomes

Ethosomes are comprised of a phospholipid bilayer membrane structure integrated with alcohol, polyglycol, and dihydrogen monooxide. These are ultra-deformable, soft, and flexible vesicular systems which have the potential to increase the permeation of bioactive into various layers of skin through topical application [59]. These vesicular systems have some additional advantages over traditional liposomes, e.g., drug penetration via epidermal layers, propensity to fluidize, and lower transition temperature of stratum corneum lipids, which could be attributable to the alcoholic content of ethosomes. Therefore, ethosomes have emerged as highly efficient drug delivery systems which have the capability to impart high drug diffusivity into deeper skin layers. It was revealed that ethosomes can increase phenylethyl resorcinol transmission across skin layers and form a drug depot inside skin with improved drug solubility and stability. It has higher inhibitory capability against tyrosinase than liposome-based formulations [60].

### 5.5. Niosomes

Niosomes are comprised of unilamellar or multilamellar vesicles made up of alkyl or dialkylpolyglycerol ethers with or without cholesterol having size range from 10–100 nm [61]. Niosomes are osmotically active vesicles with rigid membrane bilayer which offers several benefits as compared to conventional liposomes like cost effectiveness, greater chemical stability and prevention of drug leakage [61,62]. Numerous studies have reported that the topical delivery of niosomes has higher effectiveness owing to their capability to modify the drug withholding time in the stratum corneum and tendency to reduce systemic absorption [63]. The main ingredient of niosomes is nonionic surfactant which acts as permeation enhancer. The niosomes formulation of kojic acid and hydroquinone showed sustained drug delivery profiles [64]. The skin whitening effect of phenylethyl resorcinol was improved through niosomes based formulation due to an increase in skin penetration potential [65].

### 5.6. Transferosomes

Transferosomes are newly developed, elastic, and ultra-deformable vesicular systems comprised of phospholipids, water, surfactants, and a bilayer membrane softening components to enhance transdermal delivery [66]. These are highly deformable vesicles which contain edge activator like tween 80 or sodium deoxycholate in bilayer which have propensity to promote lipid bilayer flexibility and overcome obstacles to skin permeation by squeezing themselves easily across stratum corneum [67]. Linoleic acid was loaded into transferosomes which considerably improved its skin permeability in comparison to hydro-alcoholic solution [68]. Transferosomes loaded with phenylethyl resorcinol improved its solubility, stability, permeability, skin irritation, and tyrosinase inhibitory action as compared to liposomes [69]. In 2018, Limsuwan and his colleagues investigated that transferosomes demonstrated improved solubility and stability of phenylethyl resorcinol [70]. The niacinamide loaded transferosomes improved skin permeability, solubility, stability, and whitening efficacy in comparison to regular liposomes [71]. In another research, it was investigated whether arbutin-loaded transferosomes exhibited improved skin permeation and the deposition of hydrophilic skin-whitening compounds [72].

### 5.7. Aspasomes

Ascorbyl palmitate vesicles called aspasomes are composed of ascorbyl palmitate bilayer rather than phospholipids moieties present in conventional liposomes [73]. In addition, ascorbyl palmitate is an amphiphilic molecule having antioxidant action. The addition of magnesium ascorbyl phosphate to aspasomes tends to amplify epidermal permeation and retention in comparison to 15% trichloroacetic acid [74].

### 5.8. Invasomes

Invasomes are ethanol and terpene-containing vesicular systems which act as skin penetration boosters due to their capacity to disrupt the order of stratum corneum packing by assigning a net negative surface charge and inhibiting vesicle coalescence due to electrostatic repulsion force [75,76]. Terpenes are naturally occurring volatile oils that are usually regarded as a safe chemical with a minimal irritation risk at low doses (1–5%) and produce reversible influence on stratum corneum lipid deposition. It was discovered that ethanol and terpene produced a synergistic effect on percutaneous absorption [77]. A research study evaluated the transferosomes, invasomes, and conventional liposomes as skin permeation vesicular carriers of phenylethyl resorcinol and disclosed that invasomes exhibited deeper skin penetration which could be attributed to their greater deformability as compared to other two vesicles [78].

### 5.9. Penetration-Enhancing Vesicles

Penetration-enhancing vesicles (PEVs) are called absorption promoters and sorption accelerants due to presence of penetration enhancers into phospholipids bilayer [79]. PEVs are vesicular systems which have applications in improving drug penetration through the skin layers and function in similar fashion as edge activators in transferosomes [80]. PEVs, upon contact with skin keratin, tend to alter stratum corneum fluidity which ultimately results in an increase in drug partitioning across the stratum corneum [81]. The examples of penetration enhancers used in PEVs are fatty acids (e.g., stearic acid, lauric acid), capryl-caproyl macrogol 8-glyceride (Labrasol^®^), diethylene glycol monoethyl ether (Transcutol^®^), and ether alcohols (e.g., L-menthol, cineole and limonene) [82]. A group of researchers investigated the effects of lauroylcholine chloride and monoolein as penetration enhancer in the topical delivery of 3-hydroxycoumarin and revealed enhanced drug transportation to deeper skin layers [83].

## 6. Role of Inorganic Nanocarriers in Hyperpigmentation

The nanocarriers based on non-metal elements such as sulfur and diamonds are being studied for application in drug delivery for management of melasma [84]. The inorganic nanocarriers can be comprised of metal or non-metal element. The non-metal elements, e.g., diamond and sulfur, are contained within the core, while metals elements are contained inside shell and provides suitable substrate for conjugation with bio-macromolecules [85]. Inorganic nanocarriers offer superior mechanical strength, chemical stability, and controlled drug release across skin via topical drug delivery. The different types of inorganic nanocarriers are fullerenes, carbon nanotubes, iron oxide, gold nanoparticles, silver nanoparticles, and quantum dots [31].

### 6.1. Gold Nanoparticles

Gold nanoparticles (AuNPs) are small gold particles which have a high degree of stability when disseminated in water. AuNPs have characteristics, such as chemical inertness, excellent biological compatibility, and high stability [86]. In a research study, the efficacy of *Panax ginseng* leaves extract based AuNPs on tyrosinase activity, tyrosinase gene-expression, and cellular melanin concentration in murine melanoma B16BL6 cell lines was investigated. It was found that synthesized AuNPs have the potential to limit the activity of tyrosinase in B16 cells stimulated by melanocyte-stimulating hormone and suppress tyrosinase at transcriptional level in comparison to arbutin, which demonstrated their efficacy in skin whitening and depletion of melanin content [87].

### 6.2. Fullerenes

Fullerenes are spheroid ball-shaped nanoparticles with carbon structure. These are also called cylinder carbon nanotube C60 [33]. Their enormous interior capacity allows for the incorporation of bio-molecules and their outside surface can be chemically altered for topical drug delivery for management of skin disorders [88]. These are currently utilized in cosmetics as anti-aging, skin brightening, ultraviolet protection, and antioxidants for the prevention of melanogenesis progression [89]. The fullerene derivative encapsulated in hydrophilic polyvinylpyrrolidone dramatically reduced UVA-induced melanogenesis in normal human epidermal melanocytes in comparison to lascorbic acid and arbutin, which could be attributed to enhanced antityrosinase action [90]. 

## 7. Role of Polymer-Based Nanocarriers in Hyperpigmentation

Polymer-based nanocarriers are made up of biodegradable and biocompatible polymers with particle sizes ranging from 10 to 1000 nanometers [91,92]. The examples of natural polymers are chitosan, gelatin, alginate, and albumin. Synthetic biodegradable polymers include polylactide-co-glycolide and polycaprolactone, whereas synthetic non-biodegradable polymers include polymethylmethacrylate, polyacrylates and ethyl cellulose [93,94,95,96]. The polymer based nanocarriers explored for the management of hyperpigmentation include polymeric nanoparticles, polymerosomes, and polymeric micelles [97,98].

### 7.1. Polymeric Nanoparticles

The polymeric nanoparticles include either nanospheres or nanocapsules which represent a matrix or reservoir system, respectively [49]. These can be utilized for altering drug release profile, e.g., delayed release and controlled release, or their residence time in skin can be extended [99]. The drug release process from polymeric nanoparticles includes polymer swelling by hydration and drug release via diffusion, enzymatic cleavage, and drug desorption from polymeric systems [100]. The chitosan and ethyl cellulose based polymeric nanoparticles have been utilized for topical drug administration for the management of hyperpigmentation. Chitosan is a cationic, biocompatible, mucoadhesive, and FDA approved polymer for cutaneous drug delivery [101,102,103,104]. The surface coating of N-(2-hydroxyl) propyl-3-trimethyl ammonium chitosan chloride over liposomes effectively sequesters the permeability of kojic acid and data illustrated that melanin synthesis in skin was dramatically reduced in comparison to traditional liposome [105]. Ethyl cellulose is a synthetic, hydrophilic, and non-biodegradable polymer with the ability to synthesize films with excellent adherence [106]. In a research study, it was found that the incorporation of ascorbic acid into ethyl cellulose nanoparticles leads to an increase in its stability, skin diffusivity, and antityrosinase activity with improved skin whitening potential [107].

### 7.2. Polymerosomes

Polymerosomes are colloidal hollow nanospheres comprised of aqueous core and polymer-based bilayer membranes identical to lipid vesicular nanocarriers [108,109,110]. Polymerosomes have size ranging between 50 nm to 5 µm and the bilayer thickness of typical polymerosomes is approximately 10 nm, which is larger in comparison to the thickness of the lipid vesicular bilayer, which is approximately 4–5 nm [111].

### 7.3. Polymeric Micelles or Poloxamers

These core-shell nanocarriers have size range between 10 and 100 nm and have the potential to self-assemble. These are composed of amphiphilic graft copolymers generated in aqueous solutions [112]. The critical micelle concentration is the minimum concentration required by amphiphilic molecules to start micellization in order to produce polymeric micelles. The polymeric micelles have the capability to improve aqueous solubility, inhibit drug degradation, as well as increase skin hydration and drug permeability across skin layers [113]. A research study revealed that the loading of glabridin into amphiphilic cationic chitosan micelles leads to drug permeability enhancement via layers of skin and causes the inhibition of melanin production, which is required for the effective management of skin hyperpigmentation conditions [114].

## 8. Application of Nanocarriers for Management of Hyperpigmentation

The research outcomes from investigations based on nanocarriers, such as nanoemulsion, gold nanoparticles, nanospheres, dendrimers, cubosomes, carbon nanotubes, polymerosomes, liposomes, niosomes, solid lipid nanoparticles, and nanostructured lipid carrier, recently explored for topical drug delivery for the management of hyperpigmentation are compiled in Table 2.

## 9. Conclusions

Hyperpigmentation is primarily a dermatological facial ailment which manifests as a highly psychosocially relevant condition apparent as symmetrically distributed hyper-pigmented dark brown spots with serrated and irregular borders. The various outlines of pigmentation include centro-facial pattern, malar pattern, and mandibular pattern. The different factors which contribute to the development and progression of melasma include ultraviolet ray exposure, excessive application of cosmetics, photosensitizing drugs, hormonal therapy, and irregular synthesis of melanocyte stimulating hormone. This article conclusively manifested that nanotechnology-based approaches, e.g., lipid-based nanocarriers, inorganic nanocarriers, and polymer-based nanocarriers, emerge as cutting-edge technologies for encapsulating hypopigmenting molecules in order to increase their physicochemical strength and permeability into skin layers for the treatment of hyperpigmentation. The topical administration of hypopigmenting drugs based on nanotechnology could be utilized as a primary line of therapy along with the oral administration of additional therapy for the successful treatment of hyperpigmentation.

## Figures and Tables

**Table 1:** Fitzpatrick skin phototype classification system on the basis of skin texture and propensity to tan or burn.

Skin Type	Skin Texture	Aptitude to Tan
**I**	Blond/red hair, blond/red complexion, blue/green eyes	Never tan, always burn
**II**	Blond skin, blue eyes	Usually burn, difficulty in tan
**III**	White skin with a deeper hue	Average tan, early burn
**IV**	Moderate brown skin	Burns minimally, tans simply
**V**	Skin colour: dark brown	Hardly ever burns, tans very easily
**VI**	Skin colour: black	Always tans darkly and never burns

**Table 2:** The summary of research outcomes of nanocarriers based topical drug delivery explored for the management of melasma and other hyper-pigmentation disorders.

Drug (Technique)	Excipients	Outcome & Significance	Ref.
Solid Lipid Nanoparticles			
Kojic acid (high speed homogenisation and ultra-probe sonication method)	Cholesterol, Glyceryl monostearate, Tween 20, Span 60	Enhanced the cutaneous delivery of Kojic acid with increased concentration and controlled drug release into deeper skin layers	[115]
Econazole nitrate (High-shear homogenization method)	Precirol ATO 5, Tween 80	Increased permeation of drug within 1 h of its application and possessed better penetration of drug into deeper layers of skin after 3 h	[116]
Miconazole nitrate (High-pressure homogenization)	Compritol 888, Propylene glycol, Tween 80, Glyceryl monostearate	Improved skin targeting effect and accumulative absorption of drug in the skin	[117]
N-Acetyl-D-Glucosamine (High shear homogenization)	Cetyl Palmitate, Phosphatidylcholine, Hydrogenated Castor Oil	Improvement of skin hydration and elasticity in several skin disorders	[118]
Hydroquinone (Hot melt homogenization method)	Poloxamer 407, Glycerol Palmitostearate	Excellent physicochemical stability and improved drug localisation in the skin	[119]
Tyrosinase inhibitor-(Z)-5-(2,4-dihydroxy benzylidene)thiazolidine-2,4-dione (MHY498) (o/w emulsion solvent-evaporation method)	Compritol 888 ATO, Phosphatidylcholine, Poloxamer 188	MHY-SLNs exhibited prolonged release and increased skin permeation and effectively prevented UVB-induced melanogenesis	[120]
Curcumin (Pre-emulsion technique followed by ultrasonic probe sonication method)	Precirol ATO5, Tween-80	Curcumin-SLN exhibited controlled drug release up to 24 h. It showed potential for skin targeting and has potential in skin depigmentation	[121]
Melinjo (*Gnetum gnemon* L.) seed extract (High-shear homogenization and hot-melted technique)	Glyceryl monostearate, Brij CS25	Melinjo seed extract produced skin whitening effect without causing irritation	[122]
**Nanosponges**			
Azelaic acid (Melt method)	β-Cyclodextrin, diphenyl carbonate	Beta cyclodextrin nanosponges increased the solubility and depigmenting action of Azelaic acid via antioxidant and antityrosinase effects	[123]
**Polymeric Nanoparticles**			
Alpha-arbutin (Ionic gelation method)	Chitosan, tripolyphosphate sodium salt	Loading of α-arbutin into chitosan produced significant higher entrapment efficiency as compared to α-arbutin loading into tripolyphosphate	[124]
Alpha-arbutin (Ionic gelation method)	Chitosan, tripolyphosphate sodium salt, hyaluronic acid, collagen	Alpha-arbutin loaded chitosan nanoparticles hydrogels revealed better therapeutic effectiveness in melasma as compared to free drug hydrogels and also exhibited improved drug deposition into deep skin layers	[125]
Vitamin C (Modified solvent evaporation technique)	Ethyl cellulose, Pluronic F127	Polymeric nanoparticles showed sustained release behaviour till 8 h and significantly improved the therapeutic response and decreased adverse effects	[107]
Glabridin (Pressure homogenization method)	Partially myristoylated chitosan pyrrolidone carboxylate (PMCP), Polyquaternium-64, Tween 60, Butylene glycol, Cetyl ethylhexanoate,	PMCP was found efficient transdermal drug carrier for enhancing the permeation of Glabridin into epidermis of skin and suppressed the synthesis of melanin in skin	[114]
**Aspasomes**			
Mg ascorbyl phosphate (Film hydration method)	Lecithin, cholesterol	Aspasome based cream exhibited enhanced drug permeation and skin retention and showed clinical effectiveness in melasma equivalent to 15% trichloroacetic acid	[74]
**Transferosomes**			
Ascorbic palmitate (Thin-film hydration method)	Soybean phosphatidylcholine, Sodium deoxycholate	The permeation of ascorbic palmitate from transferosomes based drug delivery was higher which leads to 14.1-fold increase in ascorbic palmitate accumulation in epidermis in comparison to plain drug	[126]
**Ethosomes**			
Phenylethyl Resorcinol (Thin-film hydration method)	Soybean phosphatidylcholine, Cholesterol	Ethosomes showed increased tyrosinase inhibition activity and also decreased melatonin content as compared to other formulations in B16 melanoma cells	[60]
**Niosomes**			
Arbutin (Ultrasonic technique)	Cholesterol, Tween 20, Span 20	Research study illustrated higher drug deposition in skin layers and revealed no signs of cytotoxicity in *in-vitro* cytotoxicity test and non-irritancy on Wistar rats	[127]
**Nanostructured Lipid Carrier**			
Trans-Resveratrol (High shear homogenization technique)	Glyceryl behenate, Poloxamer 407, PEG-40 stearate, Castor oil, Caprylic/capric triglycerides	NLCs developed with PEG-40 stearate leads to 1.31 and 1.83-fold higher tyrosinase inhibition as compared to NLCs prepared with glyceryl behenate and plain trans-resveratrol solution	[128]
Hydroquinone (Homogenization emulsification method)	Sodium hydrogen sulfite, Bees wax, Caprylic/capric triglyceride, Lecithin, Span 80	Hydroquinone loaded in NLC showed enhanced permeability, improved light stability and exhibited higher tyrosinase inhibition rate	[129]
Deoxyarbutin (High-shear homogenisation and ultrasonication)	Cetyl palmitate, Myristyl myristate, Poloxamer 188, PEG-400, Sodium sulfite	Increased efficacy of deoxyarbutin to inhibit tyrosinase activity during melanogenesis in skin	[130]
Phenylethyl Resorcinol (Hot-melted ultrasonic method)	Glyceryl monostearate, Olive oil, Lecithin, Tween 80, Polyvinyl alcohol	Phenylethyl Resorcinol loaded NLCs have particle size, polydispersity index, encapsulation efficiency and loading capacity of 57.9 ± 1.3 nm, 0.24 ± 0.01, 93.1 ± 4.2% and 8.5 ± 0.4%, respectively and exhibited sustained release pattern	[131]
N-Acetyl Glucosamine (Hot homogenization technique)	Miglyol, Precirol, Poloxamer, Tween 80	N-Acetyl Glucosamine loaded NLCs have particle size of 190 nm, loading capacity of 9% and revealed significant decrease in melanin distribution pattern	[132]
**Liposomes**			
Niacinamide (High-pressure homogenization method)	Phosphatidylcholine, Cholesterol, Ceramide, Dipotassium glycyrrhizate	Flexible liposomes synthesized in this research demonstrated higher deformability, safety, skin permeability, and anti-melanogenesis activity in comparison to conventional liposomes	[71]
Tranexamic Acid (Fusion method)	Soybean phosphatidylcholine, Cholesterol, Propylene glycol	Immense reduction in MASI scores was observed in patients treated with 5% liposomal tranexamic acid in comparison with patients treated with 4% hydroquinone cream and no serious adverse effects were observed in patients treated with liposomes	[49]
Anthocyanin (Vaporization and dehydration-hydration of organic solution)	Lecithin, Cholesterol	Encapsulation of anthocyanin into liposomes enhanced its stability and reduced melanogenesis by inhibition of tyrosinase and suppression of protein expression of tyrosinase and microphthalmia-associated transcription factor	[52]
*Asparagus racemosus* extracts (Chloroform-film, Reverse-phase evaporation, Polyol dilution, Freeze-drying of monophase solution methods)	Lecithin, Phospholipon, Diosgenin, Cholesterol, Propylene glycol	Liposomes had particle size in range of 0.26–13.83 μm and zeta potentials of −1.5 to −39.3 mV. The liposomes prepared by polyol dilution containing lecithin had maximum entrapment efficiency and in-vitro tyrosinase inhibitory activity of 69.08% and 25%, respectively	[55]
Phenylethyl Resorcinol (Injection method)	Soybean lecithin, Tween 80	Liposomes had particle size in range of 160∼170 nm, drug loading of 2.45 ± 0.03% and had excellent stability	[56]
Arbutin (Film dispersion method)	Soybean Phosphatidylcholine, Cholesterol	The deposition of arbutin in epidermis/dermis layer of skin from liposome was higher in comparison to plain arbutin	[58]
**Microemulsion**			
*Punica granatum* extract (Spontaneous emulsification technique phase titration method)	Tween 80, Propylene Glycol, Palm oil	Microemulsion revealed skin compatibility and exhibited reduction in skin melanin content in healthy male volunteers	[133]
Ascorbic acid (Hydrophilic lipophilic deviation concept)	Dioctylcyclohexane, Sorbitan monolaurate, Decylglucoside, Mineral oil	Microemulsion showed transcutaneous penetration of ascorbic acid which illustrated ascorbic acid-loaded microemulsion as suitable cosmetic for skin whitening potential	[134]
**Nanoemulsion**			
Kojic monooleate (High and low energy emulsification technique)	Lemon essential oil, Castor oil, Tween 80, Xanthan gum	Cytotoxicity assay of nanoemulsion on mouse embryonic fibroblast cell line revealed safety and suitability of formulation in cosmeceutical application	[135]
Virgin coconut oil (Condensation method)	Squalene oil, Emulium Kappa, Propylene glycol	Addition of squalene oil caused reduction in ostwald ripening and increased stability of formulation	[136]

SLN: solid lipid nanoparticles; NLC: nanostructured lipid carriers.

## Data Availability

Not applicable.

## Abbreviations

AuNPs	Gold nanoparticles
FSPT	Fitzpatrick Skin Phototype
LNPs	Lipid nanoparticles
LUVs	Large unilamellar vesicles
MASI	Melasma area and severity index
MEs	Microemulsions
MVVs	Multivesicular vesicles
NEs	Nanoemulsions
NLCs	Nanostructured lipid carriers
PEVs	Penetration enhancing vesicles
PMCP	Partially myristoylated chitosan pyrrolidone carboxylate
SLNs	Solid lipid nanoparticles
SUVs	Small unilamellar vesicles
